
JOURNAL INFORMATION
==============================
NLM Title Abbreviation: ScientificWorldJournal
Journal ID: ScientificWorldJournal
NLM Journal ID: 101131163
Journal ID: TSWJ

The Scientific World Journal

ISSN: 2356-6140
EISSN: 1537-744X
Publisher: Wiley

ARTICLE INFORMATION
==============================
PMCID: PMC6084741
DOI: 10.1100/tsw.2001.420
Article ID: 946149
Article version: 1
Subjects: Extended Abstract

Cgrp Knock-Out Mice: New Findings

Westlund High Karin
Department of Anatomy and Neurosciences, University of Texas Medical Branch, 301 University Boulevard, Galveston, TX 77555, USA
Publication date: 2001
Electronic publication date: 2001 Nov 22
Volume: 1
First page: 18
Last page: 18
Copyright: Copyright © 2001 Karin Westlund High.
Copyright year: 2001
License: This is an open access article distributed under the Creative Commons Attribution License, which permits unrestricted use, distribution, and reproduction in any medium, provided the original work is properly cited.
License URL: https://creativecommons.org/licenses/by/3.0/

==============================
4th International Meeting on Calcitonin Gene-Related Peptide (CGRP)
*TheScientificWorld* (2001) 1(S1), 18
ISSN 1532-2246; DOI 10.1100/tsw.2001.420

### CGRP KNOCK-OUT MICE: NEW FINDINGS

Karin Westlund High
Department of Anatomy and Neurosciences, University of Texas Medical Branch, 301 University Boulevard, Galveston, TX 77555

Endogenous CGRP plays an important role in the plastic neurogenic changes that occur in response to peripheral inflammatory events, including the development of nociceptive behaviors. While inflammatory pain is thought to involve a variety of transmitters released from the nerve terminals, including amino acids, substance P (SP), and calcitonin gene-related peptide (CGRP), pharmacologic treatments with peptide antagonists have not been shown to hinder readily the development of hyperalgesic nociceptive responses after induction of acute arthritis. Recent studies in our laboratory have compared nociceptive responses in mice deficient in the calcitonin/αCGRP gene (CGRP-/-) and their parent strain (wild type 129/c57). The mice deficient in the calcitonin/αCGRP gene (CGRP-/-) displayed normal responses to noxious heat stimuli. Peripheral inflammation was then induced in the rat knee joint by injection of a mixture of kaolin/carrageenan (k/c) to produce a testable secondary hyperalgesia. Nociceptive response behaviors assessed included paw withdrawal latency (PWL) to radiant heat applied to the hindpaw, and the hot plate test. The CGRP-/- mice showed no signs of secondary hyperalgesia after development of knee joint inflammation, while a significant decrease in the PWL was observed in the CGRP+/+ mice as expected. The CGRP-/- mice also showed a prolonged rather than a shortened response latency in the hot plate test 4 h after knee joint injection of k/c. Immunohistological study showed that CGRP- like immunoreactivity (CGRP-LI) was absent in the spinal cord tissues taken from the CGRP -/- mice. These results more conclusively support the idea that CGRP plays a significant role in nociceptive events that occur in response to peripheral inflammation.
